# Effect of press needle stimulation on postoperative pulmonary complications in video-assisted thoracoscopic surgery patients: a randomized controlled trial

**DOI:** 10.1080/07853890.2025.2560684

**Published:** 2025-09-15

**Authors:** Jing Tan, Yong Feng, Hui Liu, Yihu Zhou, Jiaqin Cai, Yueyi Jiang, Pengyi Li, Lianbing Gu, Zhenghuan Song

**Affiliations:** a Department of Anesthesiology, Jiangsu Cancer Hospital and Jiangsu Institute of Cancer Research and The Affiliated Cancer Hospital of Nanjing Medical University, Nanjing, People’s Republic of China; b Department of Radiology, Jiangsu Cancer Hospital and Jiangsu Institute of Cancer Research and The Affiliated Cancer Hospital of Nanjing Medical University, Nanjing, People’s Republic of China; c Nanjing Medical University, Nanjing, People’s Republic of China; d Xuzhou Medical University, Xuzhou, People’s Republic of China

**Keywords:** Press needle, video-assisted thoracoscopic surgery, postoperative pulmonary complications, postoperative pain control

## Abstract

**Background:**

Patients undergoing high-risk surgical procedures frequently experience postoperative pulmonary complications (PPCs) that impact outcomes. Acupuncture has demonstrated potential benefits during surgery. Press needle stimulation is increasingly recognized as an alternative to conventional techniques. We aimed to evaluate the effect of press needle stimulation on PPCs in patients with lung cancer undergoing video-assisted thoracoscopic surgery.

**Methods:**

Eighty-seven patients undergoing video-assisted thoracoscopic surgery were randomized into press needle (*n* = 48) or sham needle (*n* = 39) groups. The press needle group underwent bilateral stimulation of acupoints LU9 (Taiyuan), RN17 (Danzhong), LU6 (Kongzui), and BL13 (Feishu) before anesthesia and during surgery. The sham group received a placebo stimulation. The primary endpoint was the incidence of PPCs within seven days post-surgery. The secondary endpoints included cytokine levels, intraoperative respiratory mechanics, postoperative pain score, analgesic consumption, and related complications.

**Results:**

Press needle acupoint stimulation was associated with a significantly lower incidence of PPCs than the sham needle group. Perioperative data, tachycardia, cough, postoperative nausea, vomiting, and most inflammatory markers exhibited no significant differences, except for lower hypoxia-inducible factor-1*α* levels in the press needle group. Hemodynamic and blood gas analyses indicated no significant cardiovascular or respiratory differences between groups. The press needle group exhibited reduced patient-controlled analgesia (PCA) pump utilization and lower total PCA usage, indicating improved pain management.

**Conclusion:**

This study indicates that press needle stimulation lowers PPC incidence and improves postoperative analgesia in patients with lung cancer undergoing video-assisted thoracoscopic lobectomy.

## HIGHLIGHTS

- Press needle stimulation is a feasible adjunct therapy for patients undergoing video-assisted thoracoscopic surgery.
- Using press needle stimulation reduces postoperative pulmonary complications.
- Press needle stimulation allows for improved pain control.
- Press needle stimulation may be an effective intervention for improving patient outcomes.

## 1. Introduction

Video-assisted thoracic surgery (VATS) is a minimally invasive procedure that offers numerous advantages, including expedited recovery and less trauma [1]. However, during single-lung ventilation, lung collapse or inflation can lead to an imbalance in ventilation/flow ratio, increased pulmonary shunt, ischemia-reperfusion injury, and mechanical ventilation-induced lung injury [2]. These can induce postoperative pulmonary complications (PPCs) [3] and adversely affect patient survival [4,5].

Acupuncture has emerged as a promising non-pharmacological adjunctive treatment option to address significant factors that hinder post-anesthetic recovery [6]. Multiple studies have provided compelling evidence supporting its effectiveness in reducing anesthesia consumption [7] and mitigating perioperative complications [8,9]. Acupuncture, based on the Meridian Theory, operates by modulating the flow of blood and qi through the body’s meridians. This modulation is crucial for regulating homeostasis, optimizing organ function, and maintaining balanced equilibrium during the perioperative period. Previous studies have demonstrated that acupuncture therapy can significantly reduce the perioperative inflammatory response and shorten hospitalization duration [9]. Growing evidence suggests that acupuncture can effectively improve patients’ lung function and enhance their quality of life [10,11]. Press needle therapy is a minimally invasive acupoint stimulation method. Unlike traditional acupuncture, the press needle is an enhanced subcutaneous needle that utilizes a shallow needling technique [12]. Extending needle retention time reduces pain and prolongs the effectiveness of acupuncture. It is characterized by its long-lasting efficacy, safety, and convenience. Clinical studies have demonstrated the efficacy of press needle therapy in treating several diseases. Nakamura et al. applied press needle acupuncture to PC4 (Ximen), LI10 (Shousanli), SP9 (Yinlingquan), and SP6 (Sanyinjiao) in patients with depression. This technique exerted its therapeutic effects on depression by improving vagal nerve function through bottom-up neuromodulation, resulting in clinical improvement with feasibility [13,14]. In other case series studies, they stimulated at PC6 (Neiguan), HT7 (Shenmen), and ST36 (Zusanli) using an active press needle for patients with chronic heart failure and observed a significantly improved exercise tolerance and quality of life of patients after active press needle intervention [9,15]. A single-center, three-arm, randomized controlled trial using active or sham press needles to BL23 (Shenshu) for patients with lower back pain exhibited a significant effect for pain relief, and also active and sham press needles were indistinguishable for the subjects [16].

However, it remains uncertain whether press needle stimulation can improve lung function and, consequently, enhance lung compliance. In this study, press needle acupuncture was selected to stimulate bilateral LU9 (Taiyuan), RN17 (Danzhong), LU6 (Kongzui), and BL13 (Feishu) points for treatment. This study aimed to explore its effect on pulmonary complications after thoracoscopic pneumonectomy.

## 2. Methods

The study was approved by our hospital from December 2021 to December 2022 (Nanjing, China). The study was ethically approved by the Ethics Committee of Jiangsu Cancer Hospital (approval No. 2020-053) and was registered at the Chinese Clinical Trial Registry (http://www.chictr.org.cn, ChiCTR2100048410). All patients provided written informed consent to participate in the study.

### 2.1. Participants

The study recruited participants aged between 18 and 65 years who were scheduled for elective video-assisted thoracoscopic surgery for lung segmentectomy or lobectomy resection under general anesthesia. The exclusion criteria were as follows: Severely compromised lung function (predicted forced vital capacity <80% or predicted forced expiratory volume in the first second <70%), intrathoracic extensive adhesions, one-lung ventilation (OLV) duration of less than 1 h, surgical procedures lasting over 4 h, and a history of chronic pain or opioid use disorder. Individuals with severe comorbidities, including pre-existing coagulopathy, cardiovascular, hepatic, and renal diseases, as well as pre-existing infections at the selected acupoint sites, were excluded, along with those exhibiting infections at the target acupuncture site or trauma. Participants who could not articulate study questions or had engaged in another clinical trial within the last three months were also excluded from the study.

### 2.2. Randomization and blinding

The participants in this prospective, randomized, and controlled trial were randomly assigned to either the press needle group (*n* = 48) or the sham needle group (the control group; *n* = 39), using opaque, sealed envelopes containing a computer-generated random schedule. The randomization process was not stratified. To minimize the potential for bias, treatment allocation was concealed from all participants, surgeons, data collectors, and statisticians throughout the study. However, the acupuncturists who administered the treatments were not blinded to the allocation.

### 2.3. Intervention procedures

The intervention procedures were conducted by an experienced acupuncturist using the Chinese national standard (GB12346-90) for acupoint locations. The participants will be blinded to the type of acupuncture treatment, and the assessor, data managers, statisticians, and study monitors will be blinded to the allocation. Patients were randomly assigned to either the press needle group or the control group. In the press needle group, the acupuncturist administered needles bilaterally at LU9 (Taiyuan), RN17 (Danzhong), LU6 (Kongzui), and BL13 (Feishu) acupoints for 30 min before anesthetic induction (Supplementary Fig. S1). This stimulation was maintained throughout the surgical procedure and continued for an additional two days post-operation. The press needles had a diameter of 0.2 mm and a length of 1.0 mm. The application of press needles does not typically elicit a deqi response; therefore, no deqi response was required. Patients in the control group received sham needle interventions at the same acupoints 30 min before anesthetic induction. The sham needles lacked a needle and were identical to the press needles in all other aspects, thereby preventing participants from predicting the allocated group solely based on their appearance. At the same defined acupuncture points, an independent acupuncturist administers either press needles or sham needles based on the group allocation. In both groups, needles were kept on the patient’s skin for 48 h postoperatively. Allocation guessing will be assessed following the final treatment. No needles were shed during the procedure. This standardized protocol aimed to ensure the consistency and reproducibility of the intervention procedures across all participants.

### 2.4. Anesthesia management

All surgeries were completed before 4:00 pm Preoperatively, penehyclidine was administered intravenously 30 min before transfer to the operating room, and external jugular vein and radial artery catheters were placed once venous access was established under local anesthesia. After pre-oxygenation for 5 min, general anesthesia was induced with midazolam (0.05 mg/kg), propofol (1.0 mg/kg), sufentanil (0.3 µg/kg), and cisatracurium (0.2 mg/kg). Three min later, patients were intubated with a single-lumen endotracheal tube guided by a bronchofiberscope for single-lung ventilation, and ventilation was performed under pressure-controlled ventilation with volume-guaranteed ventilation. We maintained the airway pressure at <30 cm H_2_O, and end-tidal arterial carbon dioxide pressure (PaCO_2_) ranged between 35 and 45 mmHg. All patients received an intravenous infusion of cis-atracurium (1–2 µg/kg/min), propofol (6–12 mg/kg/min), and remifentanil (0.1–0.2 µg/kg/min). Anesthesia was maintained to a BIS value of 40–60 and mean arterial pressure (MAP) within 20% of baseline. Atropine sulfate was administered if the heart rate declined to 50 beats/min. Tropisetron (8 mg) and Dizoxin (10 mg) were administered intravenously before surgery concluded to alleviate postoperative nausea, vomiting, and pain.

For all included patients, the surgical procedures were performed by standardized thoracoscopy by the same group of experienced surgeons. Our thoracoscopic anatomic pulmonary resection involved two ports. An incision ≤2 cm was made in the fourth or fifth intercostal space on the anterior axillary line as the working port. The thoracoscopic port was placed in the sixth or seventh intercostal space on the midaxillary line. After surgery, all patients were taken to the recovery room, and once they regained consciousness, they were transferred to the ward. Patient-controlled intravenous analgesia was administered for 48 h after surgery with a mixture of dezocine 50 mg (Yangtze River Pharmaceutical, China) and Tropisetron (Qilu Pharmaceutical, China) 4 mg diluted to 100 mL and administered at a bolus dose of 2 mL with a 15-min lockout interval and a 1-h limit of 15 mL. In the event of pain, patients were advised to repeatedly press the analgesic demand button until the pain was relieved.

### 2.5. Endpoints

The primary endpoint of this study was the incidence of postoperative lung complications within seven days after surgery. PPCs included hydrothorax, pneumonia, atelectasis, respiratory failure, and pulmonary infection, as diagnosed according to the definitions provided in a previous study [17]. The PPCs were assessed by a predetermined researcher who evaluated all patients following the definitions of PPCs. The secondary endpoints included serum concentrations of tumor necrosis factor-alpha (TNF-*α*), interleukin-1*β* (IL-1*β*), interleukin-6 (IL-6), and HIF-1*α*, relevant intraoperative respiratory mechanics indices, postoperative pain scores, analgesic dosage, PCA pump press numbers, analgesia-related complications (hypotension, nausea, vomiting, pruritus, and bradycardia), and length of postoperative hospital stay.

Postoperative pain scores were measured using a numerical rating scale (NRS) at 0, 24, 48 h, and 3 months after surgery, with 0 indicating no pain and 10 signifying severe pain. If the NRS pain score was greater than four at rest, intravenous oxycodone (10 mg; Mundipharma, Beijing, China) was administered as first-line rescue analgesia. The dose of dezocine and the same amount of morphine (dezocine: morphine = 10:10 mg) were also recorded in this study.

Serum levels of hypoxia-inducible factor 1alpha (HIF-1*α*), IL-6, and TNF-*α* were measured at T0 (30 min before anesthesia), T3 (at the end of one-lung ventilation), and T4 (48 h after surgery) using enzyme-linked immunosorbent assay.

Relevant intraoperative respiratory mechanics indices, including peak airway pressure (Ppeak), mean airway pressure (Pmean), plateau airway pressure (Pplateau), and dynamic lung compliance (Cdyn), were measured at T1 (immediately after OLV), T2 (1 h after OLV), and T3. Arterial blood gas analysis was performed to record pH and determine the partial pressure of carbon dioxide (PCO_2_) at T0, T1, T2, and T3.

### 2.6. Sample size calculation

Previously published data indicated that the incidence of PPC is 36% in patients undergoing minimally invasive lung cancer surgery [18]. Our preliminary experimental data suggested that pressing needle acupoint stimulation may reduce pulmonary complications by 15% (unpublished data). Our study required 45 patients in each group (80% power, type I error 0.05). To mitigate the risk of loss to follow-up, we increased the sample size and recruited 108 patients to ensure enough subjects for the study’s completion.

### 2.7. Statistical analysis

The Statistical Package for the Social Sciences software (version 19.0) was used for the analysis. Continuous variables conforming to normal distribution are expressed as means ± standard deviation (SD), while continuous variables not conforming to normal distribution are expressed as median (range or interquartile range (IQR)). Continuous variables conforming to normal distribution were tested with a *t*-test, while continuous variables not conforming to normal distribution were tested with the Mann–Whitney *U* test. Categorical variables were analyzed using the Chi-squared or Fisher’s exact test. Ordinal variables were analyzed using Wilcoxon rank sum tests. If the repeated measures were normally distributed, an analysis of variance was used; otherwise, a generalized estimation equation was used. Bilateral *p* < 0.05 was considered statistically significant.

## 3. Results

### 3.1. Participants’ baseline characteristics

**Figure 1.**
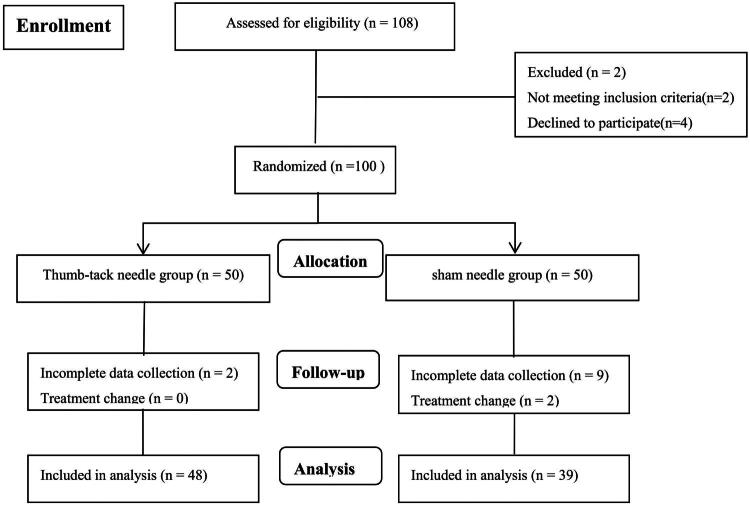
Clinical study flow chart.

Figure 1 illustrates the flowchart for patient enrollment and movement throughout the study. Initially, 108 participants were recruited for the study, of whom 100 were eligible patients. However, the final analysis included 87 participants (48 in the press needle group and 39 in the sham needle group). No significant differences were observed between the two study groups in terms of baseline patient characteristics (Table 1).

**Table 1.** Patient baseline characteristics.

Characteristics	Press needle group (*n* = 48)	Sham needle group (*n* = 39)	*P*-value
Gender (female/male)	19/29	13/26	0.548
Age (years)	60.21 (10.10)	59.38 (10.26)	0.708
Body mass index (kg/m^2^)	23.63 (2.31)	23.82 (2.89)	0.739
ASA rating (n(%))			0.927
II	30 (62.50)	24 (61.54)	
III	18 (37.50)	15 (38.46)	
Hypertension (n(%))	22 (45.83)	15 (38.46)	0.489
Diabetic (n(%))	3 (6.25)	5 (12.82)	0.292
Heart disease (n(%))	1 (2.08)	1 (2.56)	0.882
Brain stem (n(%))	3 (6.25)	1 (2.56)	0.414
Smoking status (n(%))	5 (10.42)	1 (2.56)	0.151
Drinking status (n(%))	3 (6.25)	1 (2.56)	0.414
Chemotherapy (n(%))	1 (2.08)	2 (5.13)	0.439
Operation time (min)	107.85 (42.66)	113.77 (45.74)	0.535
The procedure (n(%))			0.687
Lobectomy	30 (62.50)	26 (66.67)	
Segmentectomy	18 (37.50)	13 (33.33)	
Surgical site (n(%))			0.393
Left side	19 (39.58)	12 (30.77)	
Right side	29 (60.42)	27 (69.23)	

### 3.2. Outcome

The primary outcome of the study was the incidence of PPCs, including pneumonia, atelectasis, and respiratory failure, in the two study groups (Table 2). The findings indicated that the press needle acupoint stimulation correlated with a lower incidence of PPCs compared to the sham needle group, and the difference between the two groups was statistically significant. Specifically, 66.67% (26 patients) in the sham needle group developed PPCs, whereas only 37.50% (18 patients) in the press needle group developed PPCs, indicating a significant reduction in PPC incidence (*p* < 0.05). The most common complication observed following thoracoscopic surgery was hypoxemia, which occurred less frequently in the press needle group than in the sham needle group (*p* < 0.05). These findings suggest that press needle acupoint stimulation may be a promising approach for reducing PPCs following minimally invasive lung cancer surgery.

**Table 2.** Postoperative pulmonary complications in the first seven days after surgery.

Primary outcomes	Press needle group (*n* = 48)	Sham needle group (*n* = 39)	*P*-value
PPCs (n(%))	18 (37.50%)	26 (66.67%)	0.007*
Hydrothorax (n(%))	17 (35.42%)	8 (21.62%)	0.166
Pneumothorax (n(%))	6 (12.50%)	4 (10.53%)	0.777
Respiratory infection (n(%))	1 (2.08%)	1 (2.63%)	0.867
Atelectasis (n(%))	2 (4.17%)	0 (0.00%)	0.197
Respiratory failure (n(%))	0 (0.00%)	9 (23.08%)	< 0.001*
Pulmonary infection (n(%))	0 (0.00%)	15 (38.46%)	< 0.001*

Values are expressed as n(%). *p* < 0.05 compared with the patients in the sham needle group.

Abbreviation: PPCs, postoperative pulmonary complications including hydrothorax, pneumonia, respiratory infection, atelectasis, respiratory failure, and pulmonary infection.

### 3.3. A Comparison of perioperative data between the two groups

No significant difference was observed in the total intraoperative remifentanil consumption (*p* > 0.01) in the two groups. The duration of hospitalization after surgery was comparable between the two groups. The incidence of tachycardia, cough, and postoperative nausea and vomiting demonstrated no significant difference between the two groups (Table 3).

**Table 3.** Comparison of the two groups’ perioperative data.

	Press needle group (*n* = 48)	Sham needle group (*n* = 39)	*P*-value
Intraoperative period			
Total remifentanil amount (mg)	125.08 (41.04)	133.55 (45.59)	0.365
Total fluid (mL)	1500.00 (331.98)	1423.08 (293.31)	0.261
Intraoperative bleeding (mL)	73.12 (52.27)	44.10 (26.53)	0.002
Postoperative period			
Postoperative hospital stays (days)	6.12 (2.51)	5.82 (1.48)	0.506
Total analgesic amount (mg)			
Nausea	1 (2.08%)	0 (0.00%)	0.365
Vomiting	3 (6.25%)	1 (2.56%)	0.414
Hypotension	1 (2.08%)	1 (2.56%)	0.882
Dizzy	1 (2.08%)	1 (2.56%)	0.882
Weakness	0 (0.00%)	2 (5.13%)	0.112

### 3.4. Concentrations of TNF-α, IL-1, IL-6, and HIF-1α in the serum

**Figure 2.**
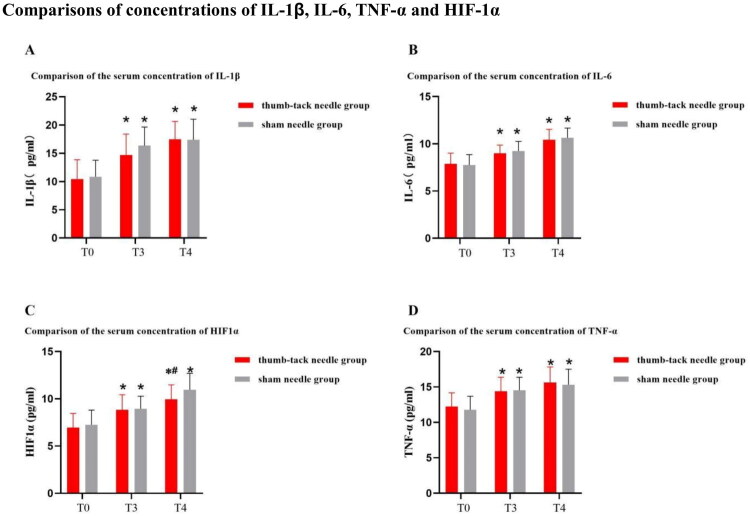
Comparisons of concentrations of IL-1*β*, IL-6, TNF-*α* and HIF-1*α* between thumb-tack needle group and con group at different time course. Abbreviation:IL-1ß, interleukin-1ß; IL-6, interleukin-6; TNF-*α*, tumor necrosis factor-*α*; HIF-1*α*, hypoxia-inducible factor- 1*α*; T0, 10 min before intubation; T3, at the end of one-lung ventilation; T4, 24h after surgery. #*p* < 0.05, vs. T0; *p < 0.05, vs. con group.

Both groups exhibited a significant increase in plasma concentrations of TNF-*α*, IL-1, IL-6, and HIF-1*α* serum levels at T3 and T4 (*p* < 0.05), as compared to the pre-anesthesia baseline. However, no significant differences were observed in the levels of IL-1*β*, IL-6, and TNF-*α* between the press needle group and the sham needle group. At T4, the HIF-1*α* serum levels in the press needle group were significantly lower than those in the sham needle group (*p* < 0.05). These findings suggest that the use of a press needle did not induce any additional pro-inflammatory response compared to the sham needle but may influence HIF-1*α* levels (Figure 2).

### 3.5. Hemodynamics and blood gas analysis

**Figure 3.**
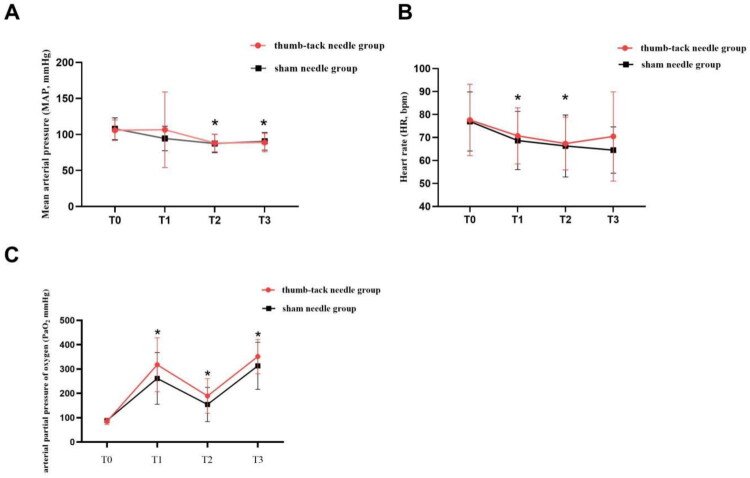
Comparison of heart rate, mean arterial pressure and arterial partial pressure between the two groups at each time point. (A) Mean arterial pressure. (B) Heart rate. (C) Arterial partial pressure. The patients in the thumb-tack needle group received acupuncture stimulation of bilateralbilateral LU9 (taiyuan), RN17 (shanzhong), LU6 (kongzui) and BL13 (feishu) acupoints from 30 min before anesthesia to the end of surgery. The patients in the control group were given an intervention in the same acupoints with sham needle. T0, 10 min before intubation; T1, one-lung ventilation for 10 min; T2 one-lung ventilation for 1 h; T3, at the end of one-lung ventilation. The data is presented as a mean ± standard deviation (x ± s). *p < 0.05 versus T0; ^#^*p* < 0.05 versus the sham needle group.

No significant differences were observed in MAP and heart rate between the two groups (*p* > 0.05; Figure 3A and B). Blood gas analysis (Table 4) revealed that changes in arterial pH and PaCO_2_ over time were similar in both groups. However, a significant decrease in oxygen partial pressure (PaO_2_) was observed after 30 min of one-lung ventilation in both groups at T2. The sham needle group exhibited greater deterioration in PaO_2_ than the press needle group. However, this difference did not reach statistical significance (Figure 3C). Other measures of intraoperative respiratory parameters were also similar between both groups (Table 4). These results suggest that the use of a press needle had no significant impact on cardiovascular or respiratory parameters compared to the sham needle.

**Table 4.** The intraoperative respiratory parameters.

Parameters	Group	T0	T1	T2	T3
pH	Press needle group	7.44 ± 0.03	7.35 ± 0.05	7.34 ± 0.05	7.35 ± 0.06
	Sham needle group	7.43 ± 0.04	7.35 ± 0.04	7.33 ± 0.06	7.34 ± 0.06
SpO_2_ (%)	Press needle group	98.33 ± 1.68	98.79 ± 3.30	98.87 ± 1.69	99.62 ± 0.63
	Sham needle group	98.46 ± 1.37	98.87 ± 1.58	97.82 ± 2.54	99.62 ± 0.59
PaO_2_ (mmHg)	Press needle group	84.79 ± 12.60	317.65 ± 110.69*	189.54 ± 71.34*	351.54 ± 70.70*
	Sham needle group	88.49 ± 8.89	261.56 ± 106.52	154.23 ± 70.48	313.21 ± 96.71
PaCO_2_ (mmHg)	Press needle group	38.88 ± 3.24	47.46 ± 6.16	48.12 ± 4.77	47.23 ± 6.34
	Sham needle group	38.21 ± 3.99	46.23 ± 6.12	48.72 ± 6.44	47.26 ± 6.79
Ppeak	Press needle group	–	17.85 ± 3.38	18.74 ± 3.40	14.29 ± 2.66
	Sham needle group	–	18.69 ± 3.45	18.95 ± 3.21	19.41 ± 33.21
Pplat	Press needle group	–	16.83 ± 3.26	17.69 ± 3.32	16.50 ± 11.19
	Sham needle group	–	16.21 ± 5.14	16.21 ± 5.14	12.08 ± 3.81
COM	Press needle group	–	29.04 ± 7.39*	28.10 ± 9.23	47.04 ± 12.25
	Sham needle group	–	25.85 ± 5.74	25.97 ± 8.68	47.69 ± 11.99

Continuous variables are expressed as mean ± SD, and intergroup comparisons are performed using two independent samples t-tests. T0, previous night of the surgery; T1, one-lung ventilation for 10 min; T2, one-lung ventilation for 1 h.

T3, at the end of one-lung ventilation.

Abbreviation: SpO_2_, saturation of pulse oxygen; PaO_2_, arterial O_2_ partial pressure; PaCO_2_, carbon dioxide partial pressure; Ppeak, peak airway pressure; Pplat, plateau pressure; COM, lung compliance.

### 3.6. NRS score

**Figure 4.**
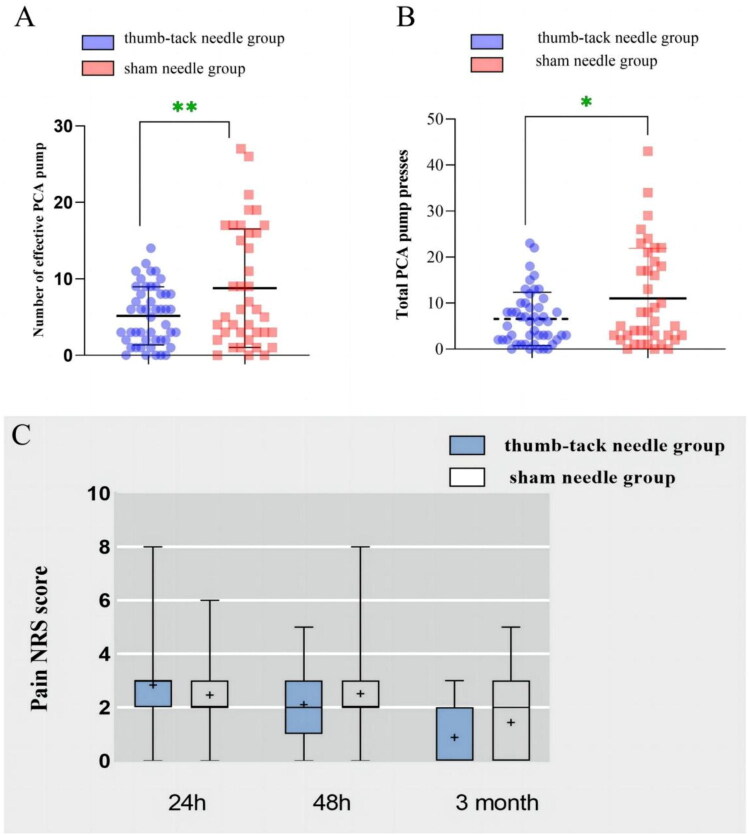
Postoperative PCA evaluation between the thumbtack needle group and sham needle group. (A) comparison of effective PCA attempts; (B) Comparison of total PCA attempts; (C) Pain NRS distribution in both the groups at 24h, 48 h, and 3 months after surgery. PCA, patient controlled intravenous analgesia. NRS, numerical rating scale. *p < 0.01, **p < 0.001.

Using press needle therapy post-operation resulted in a reduction in the utilization of effective PCA pumps in patients compared to the control group (Figure 4A). Moreover, the press needle group had a lower total number of PCA pump uses (Figure 4B; *p* < 0.01). No significant differences (*p* > 0.05) were observed in the preoperative NRS scores between the two groups (Figure 4C).

## 4. Discussion

Our studies substantiate that press needle acupuncture is an effective technique for preventing PPCs in patients with VATS. The investigation revealed that pulmonary complications occurred in both treatment options after surgery; however, the incidence of PPCs was lower in the press needle group than in the control group. The press needle group also exhibited reduced analgesic presses and enhanced analgesic efficacy.

PPCs present a significant challenge for patients undergoing lung cancer surgery, and currently available treatments are insufficient. An important factor contributing to PPCs is the use of single-lung mechanical ventilation in the lateral supine position during thoracic surgery, which can result in lung collapse and retention [3] as well as alveolar inflammation. This imbalance between pro-inflammatory and anti-inflammatory responses can cause pathophysiological changes, including vascular endothelial cell damage, increased capillary permeability, and platelet adhesion. Moreover, the stress of surgery can disrupt the brain’s neuroendocrine system, hormones, cytokines, and immune system, leading to inflammatory reactions [19] and further exacerbating the risk of PPCs. These complications can manifest as atelectasis, pneumonia, or pleural effusion, among others [20]. Respiratory failure was defined as a SpO_2_ of less than 90% or a PaO_2_ of less than 60 mmHg or the need for non-invasive ventilation or intubation with invasive mechanical ventilation [21].

Acupuncture has garnered increasing attention in recent years for its potential to improve lung function and reduce postoperative acute lung injury [22]. Researchers have focused significantly on the subject, with studies suggesting that acupuncture can enhance the activity of mitochondrial respiratory enzymes by regulating the oxidative stress response, thereby improving respiratory chain function [23]. Clinical observations have indicated that acupuncture can improve pulmonary function and enhance the quality of life for patients [10,11]. Patients with asthma and chronic obstructive pulmonary disease have demonstrated improvement after acupuncture treatment [24,25]. The press needle technique is a type of acupuncture that involves inserting a needle into the skin for an extended period to treat various ailments [26]. This technique offers several benefits, including minimal invasiveness, ease of operation, and greater patient tolerance.

Traditional Chinese medicine theory posits that the stimulation of the pulmonary system is the principal cause of perioperative pulmonary complications. The acupoints located on the bladder meridian line of the foot-Taiyang, which correspond to the lungs, are essential locations where lung qi is transferred and replenished. These points are crucial for treating respiratory disorders. Animal experiments have demonstrated that stimulating the BL13 (Feishu) and ST36 (Zusanli) acupoints in rabbits can reduce the aggregation of inflammatory cells, including neutrophils, and decrease the release of inflammatory mediators, thus alleviating lung injury caused by endotoxin shock [27]. Clinical studies have demonstrated the effectiveness of acupuncture in addressing respiratory disorders. In lung cancer resection, patients may experience an imbalance in the ventilation/blood flow ratio, leading to hypoxemia and impaired lung function [28]. Acupuncture can enhance the ventilation/perfusion ratio [29], reducing the rate of pulmonary shunt and promoting pulmonary oxygenation [30].

In this study, we selected Feishu (BL13), LU9 (Taiyuan), RN17 (Shanzhong), and LU6 (Kongzui) as acupoints based on prior literature. Liu et al. [31] reported that preoperative 30-min stimulation of BL13 (Feishu) and ST36 (Zusanli) in patients undergoing radical resection of lung cancer effectively reduced postoperative inflammatory reactions and pulmonary complications. Acupuncture at BL13 has been demonstrated to improve pulmonary function and reduce inflammation in lung tissue and cells in previous studies [32,33]. Moreover, BL13 (Feishu) is frequently employed in the treatment of respiratory conditions, including cough, asthma, and chest tightness [34,35].

In our study, the observed incidence of complications appears to be higher than initially anticipated. Multiple plausible explanations may elucidate this fact. First, the limited sample size restricted our ability to investigate the associations between press needle stimulation and PPCs thoroughly. Some sham group patients demonstrated suboptimal postoperative adherence and self-removing press needle devices, posing an unexpected challenge to data completeness. The higher incidence of PPCs may have necessitated increased nursing care and movement, potentially leading to detachment of the needle apparatus. Consequently, data loss occurred in nine patients, resulting in an insufficient sample size for the sham group, which may have impacted the reliability of the conclusions. Moreover, the prevalence of hydrothorax, identified as the predominant respiratory complication in our study, differed from another study, which indicated pneumothorax as the most prevalent pulmonary complication. Furthermore, patient-related risk factors, including age, hypertension, nutritional status, ASA classifications, and smoking, were identified, and these factors are considered plausible mechanisms that could explain the elevated incidence of pulmonary complications in our patient population. However, our study revealed a significant reduction in the incidence of PPs during the perioperative period in the press needle group compared to the sham needle group. One possibility is that the sham procedure itself may have unintended physiological effects or induce inflammatory responses, which could lead to the observed higher PPC rates. However, our results indicate that the press needle group, which underwent a procedure similar to the sham group but with actual needles, did not exhibit a similarly elevated PPC incidence. This suggests that the use of press needles, compared to the sham procedure, does not induce any additional pro-inflammatory reactions. The instruments used in the sham group lacked actual needles and were expected to exert no discernible impact on the patients. Consequently, based on our findings, we do not believe that the sham procedure contributed to elevated PPC rates. Moreover, patients who underwent press needle acupuncture exhibited a trend towards elevated PaO_2_ levels compared to those who received sham needle treatment, indicating improved oxygenation and pulmonary diffusion function. These results suggest that acupuncture may contribute positively to reducing the imbalance in the ventilation/blood flow ratio during OLV and improving respiratory outcomes in patients undergoing thoracic surgery [29,36].

Acupuncture has been demonstrated to be effective through several mechanisms. First, acupuncture can regulate immune function through the cholinergic anti-inflammatory pathway, which has a bidirectional regulatory effect on the body’s immune state [37]. Acupuncture can also inhibit the inflammatory response, rendering it a promising therapeutic alternative for inflammatory disorders [38,39]. Second, acupuncture activates the hypothalamus-pituitary-adrenal axis and inhibits the synthesis of pro-inflammatory cytokines through the vagus nerve in a hormone-like manner. This mechanism provides a possible explanation for the anti-inflammatory effects of acupuncture, suggesting that it may be beneficial for addressing inflammatory disorders, including respiratory diseases [40]. Third, acupuncture has been demonstrated to positively impact dyspnea by enhancing the release of endogenous opioids and regulating the limbic system. Acupuncture can provide an anti-inflammatory effect by promoting the chemokine-mediated proliferation of opioid-containing macrophages in inflammatory tissues. These mechanisms suggest that acupuncture possesses significant potential as a complementary therapy for improving respiratory symptoms and reducing inflammation [10,41].

This study revealed that serum concentrations of IL-1, IL-6, HIF1*α*, and TNF-*α* increased in both groups at the end of one-lung ventilation and 48 h after surgery. However, compared to the control group, the HIF1*α* concentration decreased at the end of one-lung ventilation after press-needle treatment. TNF-*α*, IL-1*β*, and IL-6 are well-known pro-inflammatory cytokines, and HIF1*α* is a critical regulator of inflammatory responses [42,43]. Previous studies have demonstrated that single-lung ventilation may induce oxidative stress and an inflammatory response, leading to lung injury. However, the press needle treatment on acupoints such as the pulmonary point may regulate the level of inflammatory factors and achieve a stable and balanced state [44,45]. This can reduce the oxidative stress injury and inflammatory response induced by single-lung ventilation, thereby protecting the lung [46]. Moreover, acupuncture therapy is based on self-regulation of the human body, and press needle therapy may improve local blood circulation by regulating the balance between endothelial vasoconstriction and dilation. This can restore the physiological balance state and adjust the internal environmental homeostasis [47]. Moreover, both groups might benefit from using protective ventilation strategies during surgery to improve oxygenation [48]. Clinical studies have suggested that protective ventilation strategies can reduce the over-expression of pro-inflammatory factors, reduce the degree of lung injury, and lower the incidence of PPCs [49,50].

Press needle treatment may also effectively improve postoperative pain symptoms despite no significant differences in the NRS scores between the two groups. Our data revealed that the press needle significantly decreased both the effective and total patient-controlled intravenous analgesia attempts compared with the sham group, implying that the press needle might exert an analgesic effect. Previous studies have extensively investigated the analgesic effects of acupuncture, which may be attributed to its ability to enhance the release and effect of neurotransmitters and hormones, including opioids, serotonin, adenosine, and signal molecules [51–53].

Poor postoperative pain control after video-assisted thoracoscopic surgery can lead to chronic post-surgical pain over time and adversely affect respiratory mechanics, potentially increasing the risk of PPCs [54]. Guidelines for lung surgery strongly recommend opioid-sparing analgesia control to improve the quality of enhanced recovery after surgery (ERAS) [55]. Managing acute postoperative pain enables patients to engage in exercises that enhance pulmonary function, a crucial element in promoting pulmonary function recovery [56]. As demonstrated in the study, the use of a press needle has been highly effective in reducing pain both immediately after its application and after a longer course of treatment. This may also have affected the rate of PPCs. Consequently, the press needle may be a useful tool for managing postoperative pain in patients undergoing VATS. These findings suggest that the press needle can be a valuable addition to pain management strategies in the context of ERAS protocols.

This study has some limitations. This study employed a single-center design; a larger multicenter study with a prospective design is necessary to enhance the generalizability of the findings. Additionally, the fact that fewer lobectomies were performed in the press needle group than in the sham needle group may have influenced PPCs. The small sample size and short follow-up period limited the ability to determine the long-term effects of press needle treatment on PPCs and pain patterns. Despite these limitations, this study has several valuable implications for future research. By integrating a sham acupuncture group and meticulously delineating the inclusion criteria, we excluded surgical procedures that varied significantly in scope, trauma severity, and postoperative recovery time factors that could substantially influence the incidence and type of PPCs. These factors contribute to reducing bias risks and provide important insights into the effects of press needle stimulation combined with patient-controlled analgesia on PPCs and pain levels in patients undergoing thoracoscopic lung surgery. Future studies should address the limitations of this study and investigate the long-term effects of press-needle therapy in greater detail.

## 5. Conclusion

Our study demonstrated a correlation between the use of press needle acupuncture and a reduction in postoperative pain and analgesic consumption in patients undergoing minimally invasive lung surgery. This non-invasive and non-pharmacological approach may provide a simple and convenient option for addressing future clinical requirements.

## Supplementary Material

Supplementary Fig.jpg

Figure and table legends.doc

## Data Availability

The data cannot be shared publicly because of participant privacy issues. Researchers can access the data by submitting a suitable proposal to the corresponding author ZS.
