# Supplementary figures and images for: Effect of press needle stimulation on postoperative pulmonary complications in video-assisted thoracoscopic surgery patients: a randomized controlled trial
Source: Ann Med. 2025 Sep 15;57(1):2560684. doi: 10.1080/07853890.2025.2560684 (PMC12439804; doi:10.1080/07853890.2025.2560684)

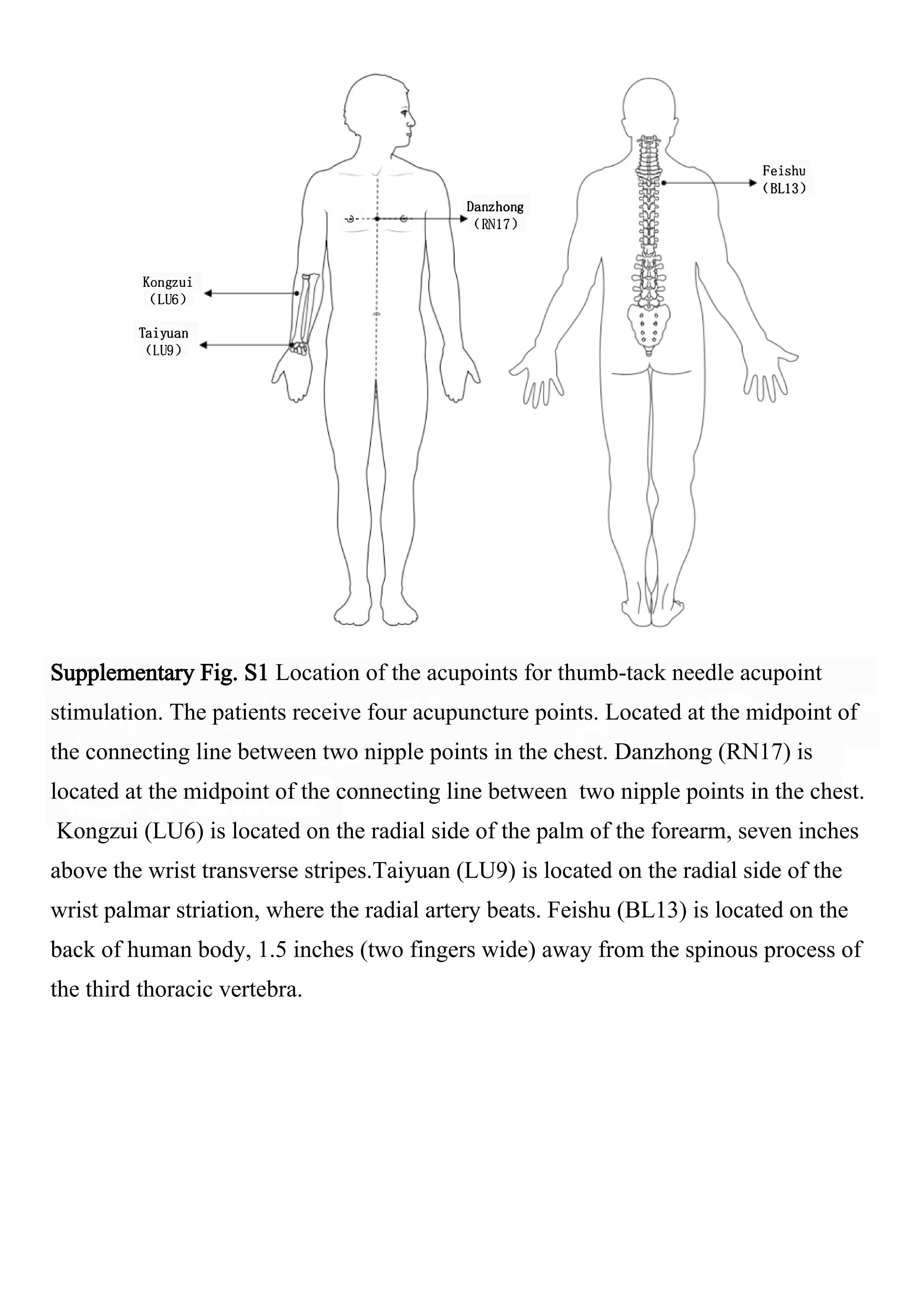

Supplement: Supplementary Fig.jpg [file IANN_A_2560684_SM8479.jpg]
