# Supplementary material for: Effect of press needle stimulation on postoperative pulmonary complications in video-assisted thoracoscopic surgery patients: a randomized controlled trial
Source: Ann Med. 2025 Sep 15;57(1):2560684. doi: 10.1080/07853890.2025.2560684 (PMC12439804; doi:10.1080/07853890.2025.2560684)
Supplement: Figure and table legends.doc [file IANN_A_2560684_SM8478.doc]

**Table 1** Patient baseline characteristics.

**Legend :** The data are presented as means (SD), or numbers (proportions).

Abbreviations: ASA: American Society of Anesthesiologists; BMI: body mass index.

**Table 2.** Postoperative pulmonary complications in the first seven days after surgery.

**Legend :**

Values are expressed as n (%). P<0.05 compared with the patients in the sham needle group.The incidence of PPCs within the postoperative 7 days was 37.50% in the thumb-tack needle group, and 66.67% in the shame needle group (P =0.007). Significacnt difference was found for the primary outcome in the thumb-tack needle group compared with that in the sham needle group. The incidence of pulmonary complications within postoperative 7 days, compared individually, thumb-tack needle group participants had a significantly lower incidence of pulmonary infections, and respiratory failure than sham needle group participants (P value for each<0.001).

Abbreviation: PPCs postoperative pulmonary complications (hydrothorax，pneumonia, atelectasis, respiratory failur and pulmonary infection.)

**Table 3** Comparison of the two groups' perioperative data

**Legend :**

The data are expressed as mean(±SD) or as frequency (percentage) n (%). P<0.05 Compared with the patients in the sham needle group.

**Table 4.** The intraoperative respiratory parameters.

**Legend :**

Continuous variables are expressed as mean± SD, and intergroup comparisons are performed using two independent samples t-tests. T0, previous night of the surgery; T1, one-lung ventilation for for 10 minutes; T2 one-lung ventilation for 1 hour; T3, at the end of one-lung ventilation. Abbreviation: SpO2, Saturation of Pulse Oxygen; PaO2, arterial O2 partial pressure; PaCO2, carbon dioxide partial pressure; Ppeak, peak airway pressure ; Pplat plateau pressure ; COM, lung compliance.

**Fig. 1** Clinical study flow chart.

**Fig. 2** Comparisons of concentrations of IL-1β, IL-6, TNF-α and HIF-1α between thumb-tack needle group and Con group at different time course. Abbreviation:IL-1ß, Interleukin-1ß; IL-6, Interleukin-6; TNF-α, Tumor necrosis factor-α; HIF-1α, Hypoxia-inducible factor- 1α; T0, 10 min before intubation; T3, at the end of one-lung ventilation; T4, 24h after surgery. #P<0.05, vs. T0;

*P<0.05, vs. Con group

**Fig. 3** Comparison of heart rate , mean arterial pressure and arterial partial pressure between the two groups at each time point. (A) Mean arterial pressure. (B) Heart rate. (C) Arterial partial pressure. The patients in the thumb-tack needle group received acupuncture stimulation of bilateralbilateral LU9 (Taiyuan), RN17 (Shanzhong), LU6 (Kongzui) and BL13 (Feishu) acupoints from 30 min before anesthesia to the end of surgery. The patients in the control group were given an intervention in the same acupoints with sham needle. T0, 10 min before intubation; T1, one-lung ventilation for 10 minutes; T2 one-lung ventilation for 1 hour; T3, at the end of one-lung ventilation. The data is presented as a mean ± standard deviation.(x ± s). ^∗^ P < 0.05 versus T0; ^#^P<0.05 versus the sham needle group.

**Fig. 4** Postoperative PCA evaluation between the thumbtack needle group and sham needle group.(A) Comparison of effective PCA attempts; (B) Comparison of total PCA attempts; (C) Pain NRS distribution in both the groups at 24h, 48 h, and 3months after surgery. PCA, patient controlled intravenous analgesia. NRS, numerical rating scale. *P < 0.01，**P < 0.001

**Supplementary Fig. S1** Location of the acupoints for thumb-tack needle acupoint stimulation. The patients receive four acupuncture points. Located at the midpoint of the connecting line between two nipple points in the chest. Danzhong (RN17) is located at the midpoint of the connecting line between two nipple points in the chest. Kongzui (LU6) is located on the radial side of the palm of the forearm, seven inches above the wrist transverse stripes.Taiyuan (LU9) is located on the radial side of the wrist palmar striation, where the radial artery beats. Feishu (BL13) is located on the back of human body, 1.5 inches (two fingers wide) away from the spinous process of the third thoracic vertebra.
